# Down-regulation of AR splice variants through XPO1 suppression contributes to the inhibition of prostate cancer progression

**DOI:** 10.18632/oncotarget.26239

**Published:** 2018-10-19

**Authors:** Amro Aboukameel, Irfana Muqbil, Erkan Baloglu, William Senapedis, Yosef Landesman, Christian Argueta, Michael Kauffman, Hua Chang, Trinayan Kashyap, Sharon Shacham, Jasper E. Neggers, Dirk Daelemans, Elisabeth I. Heath, Asfar S. Azmi

**Affiliations:** ^1^ Wayne State University School of Medicine, Detroit, MI, USA; ^2^ University of Detroit Mercy, Detroit, MI, USA; ^3^ Karyopharm Therapeutics Inc, Newton, MA, USA; ^4^ KU Leuven Department of Microbiology and Immunology, Laboratory of Virology and Chemotherapy, Rega Institute for Medical Research, Herestraat, Belgium

**Keywords:** metastatic prostate cancer, AR SPLICE variant, nuclear export, CRM1, SINE

## Abstract

Emerging studies have shown that the expression of AR splice variants (ARv) lacking ligand-binding domain is associated with castrate-resistant prostate cancer (CRPC) and higher risk of tumor metastasis and recurrence. Nuclear export protein XPO1 regulates the nuclear localization of many proteins including tumor suppressor proteins. Increased XPO1 in prostate cancer is associated with a high Gleason score and bone metastasis. In this study, we found that high expression of AR splice variant 7 (AR-v7) was correlated with increased XPO1 expression. Silencing of XPO1 by RNAi or treatment with Selective Inhibitor of Nuclear Export (SINE) compounds selinexor and eltanexor (KPT-8602) down-regulated the expression of AR, AR-v7 and ARv567es at mRNA and protein levels. XPO1 silencing also inhibited the expression of AR and ARv regulators including FOXA1, Src, Vav3, MED1 and Sam68, leading to the suppression of ARv and AR target genes, UBE2C and PSA. By targeting XPO1/ARv signaling, SINE suppressed prostate cancer (PCa) growth *in vitro* and *in vivo* and potentiated the anti-cancer activity of anti-AR agents, enzalutamide and abiraterone. Therefore, XPO1 inhibition could be a novel promising agent used in combination with conventional chemotherapeutics and AR-targeted therapy for the better treatment of PCa, especially CRPC.

## INTRODUCTION

Prostate cancer (PCa) remains the most common cancer and the second leading cause of cancer related deaths in men in the United States. The American cancer society estimated that 161,360 new cases of PCa will be diagnosed and 26,730 patients with PCa will die in 2017 [1]. The high mortality of patients with PCa is due to the development of castrate-resistant PCa (CRPC) that fails to respond to androgen deprivation therapy (ADT) and subsequently metastasizes. Growing evidence demonstrates that the molecular mechanisms involved in the development and progression of PCa and CRPC include alterations in androgen receptor (AR), Akt, Wnt, Hedgehog, and other signal transduction pathways [2–5]. One of the most important cellular signaling pathways involved in the development of PCa and CRPC is AR signaling [2]. AR is activated by androgen binding and translocates to nucleus to activate transcription of AR-target genes. It is believed that AR over-expression and androgen hypersensitivity promote enhanced nuclear translocation and activation of AR signaling, which allows CRPC to proliferate in the presence of anti-androgen therapy. Emerging studies have shown that the expression of AR splice variants (ARv), which lack a ligand-binding domain, is increased in androgen-independent prostate cancer cell lines, CRPC, and metastatic PCa [6–10]. Although lacking a ligand binding domain, these ARv are constitutively activated and their transcriptional activity is not regulated by androgens or anti-androgens [11]. ARv are also associated with CRPC and a higher risk of tumor recurrence [12, 13]. These findings suggest that prostate cancer cells expressing ARv could drive the development of the CRPC phenotype, tumor progression, recurrence and metastasis. Therefore, inhibiting ARv could be a promising strategy for the treatment of CRPC.

The nuclear export protein exportin-1 (XPO1, also known as CRM1) regulates the cellular localization of many proteins including tumor suppressor proteins (TSPs). XPO1 is overexpressed in various cancers including prostate cancer and is correlated with poor prognosis [14–16]. Moreover, increased XPO1 in prostate cancer has been found to be associated with increased Gleason score and bone metastasis [14]. It has been reported that selinexor, a Selective Inhibitor of Nuclear Export (SINE), covalently binds to the cargo binding pocket of XPO1, thus preventing the export of its many cargos. Inhibiting XPO1 mediated nuclear export with selinexor leads to an enrichment of cell cycle regulators and TSPs in the nucleus, which ultimately promotes cell cycle arrest and apoptosis [17]. Selinexor has also been found to reduce tumor spreading and improve overall survival in preclinical models of prostate cancer [18]. *In vitro*, treatment of prostate cancer cells with selinexor resulted in XPO1 inhibition, which led to the nuclear retention of p53 and Foxo proteins and the degradation of cyclin D1, survivin and XPO1, consequently triggering apoptosis [14, 19]. More significantly, in a Phase II study Selinexor demonstrated clinical activity in abiraterone- and/or enzalutamide-refractory mCRPC patients refractory to second-line anti-androgenic agents (NCT02215161) [20]. However, the detailed molecular mechanisms underlying SINE-inhibited prostate cancer growth are not clear. Whether SINE (selinexor and KPT-8602 which is a new generation SINE) could regulate important AR signaling in prostate cancer through modulation of AR and ARv remains unknown. Therefore, we investigated the *in vitro* and *in vivo* effects of SINE on the regulation of AR and ARv in prostate cancer and the molecular mechanisms underlying XPO1 regulated AR and ARv in order to design a novel therapeutic strategy for the treatment of prostate cancer and CRPC.

## RESULTS

### PCa has high expression of XPO1 mRNA and the high expression of XPO1 is correlated with AR-v7 expression

By utilizing and analyzing the mRNA microarray data in Oncomine database, we observed more than eight sets of data which showed higher expression of XPO1 mRNA in prostate cancer tissues compared to normal prostate gland tissue (Supplementary Figure 1). These results suggest that PCa cells express higher levels of XPO1, which could contribute to carcinogenesis and progression of PCa. These results are consistent with the previous report showing that the high expression of XPO1 in PCa tissue is associated with an increased Gleason score and bone metastasis [14].

**Figure 1:**
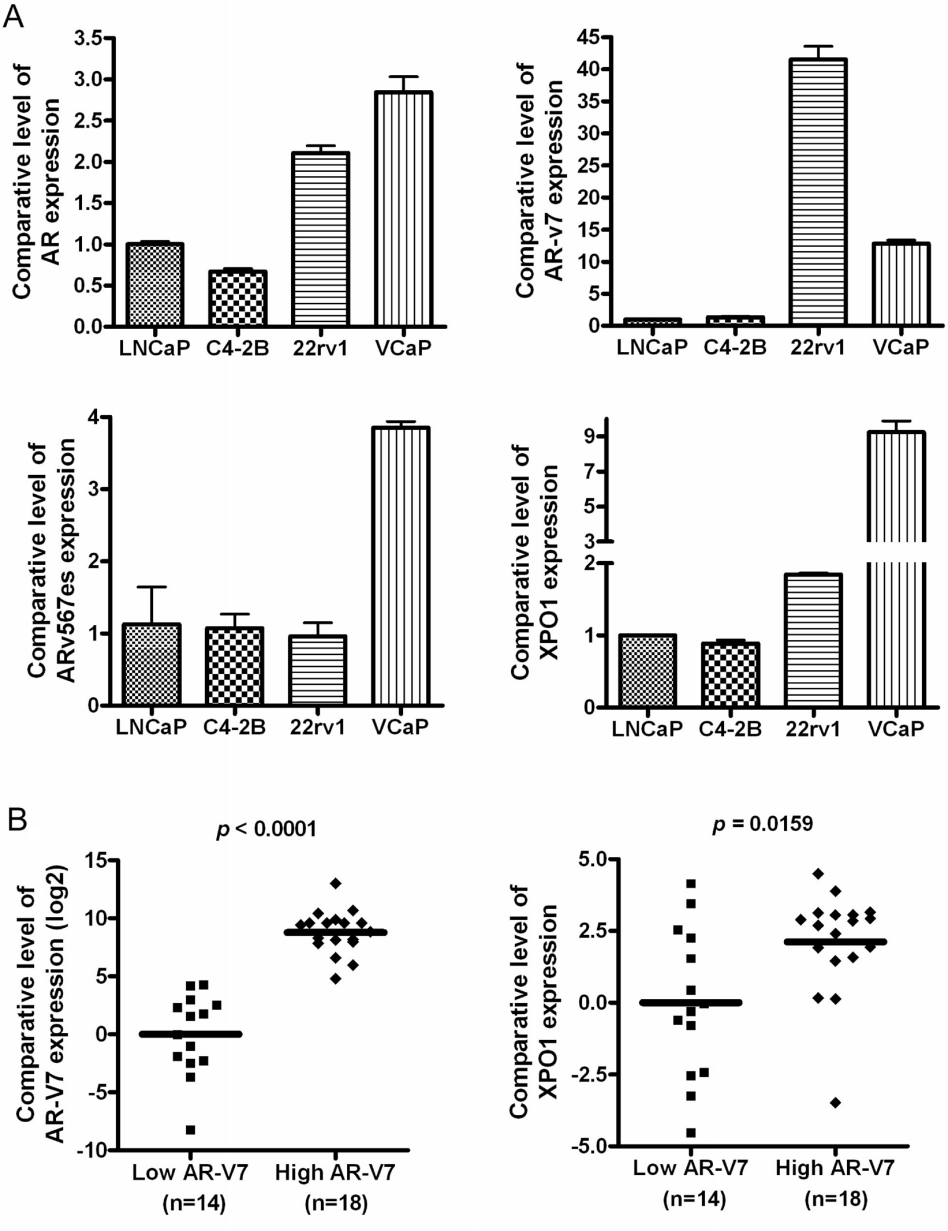
High expression of AR splice variants is correlated with over expression of XPO1 in PCa cells (**A**) The expression levels of AR, AR-v7, ARv567es and XPO1 mRNA in 22Rv1, VCaP, LNCaP and C4-2B PCa cells were measured by using real-time RT-qPCR. (**B**) The expression levels of AR-v7 and XPO1 mRNA in paraffin-embedded tissues from 32 cases of PCa patients were assessed by real-time RT-qPCR.

To determine the basal levels of AR and AR splice variants including AR-v7 and ARv567es, we conducted real-time RT-qPCR to measure the expression levels of these molecules in LNCaP, C4-2B, 22Rv1 and VCaP cells. We found that 22Rv1 and VCaP cells expressed significantly high level of AR splice variant AR-v7 compared to LNCaP and C4-2B cells and that 22Rv1 had highest expression of AR-v7 among these four cell lines (Figure 1A). In addition, VCaP cells showed much higher expression of ARv567es. Since both XPO1 and ARv are associated with progression of PCa, we tested whether there is a connection between XPO1 and ARv. We found that LNCaP and C4-2B cells exhibit lower expression levels of AR splice variants, when compared to 22Rv1 and VCaP cells which harbor high levels of AR spice variants and XPO1 (Figure 1A). Furthermore, we tested the expression levels of AR-v7 and XPO1 in tumor tissues from the patients with PCa. We found that the tumor tissues with high level of AR-v7 also expressed higher level of XPO1 (Figure 1B), suggesting that there could be a molecular interaction between XPO1 and AR splice variants.

### Silencing of XPO1 inhibits AR splice variants and their regulators

**Figure 2:**
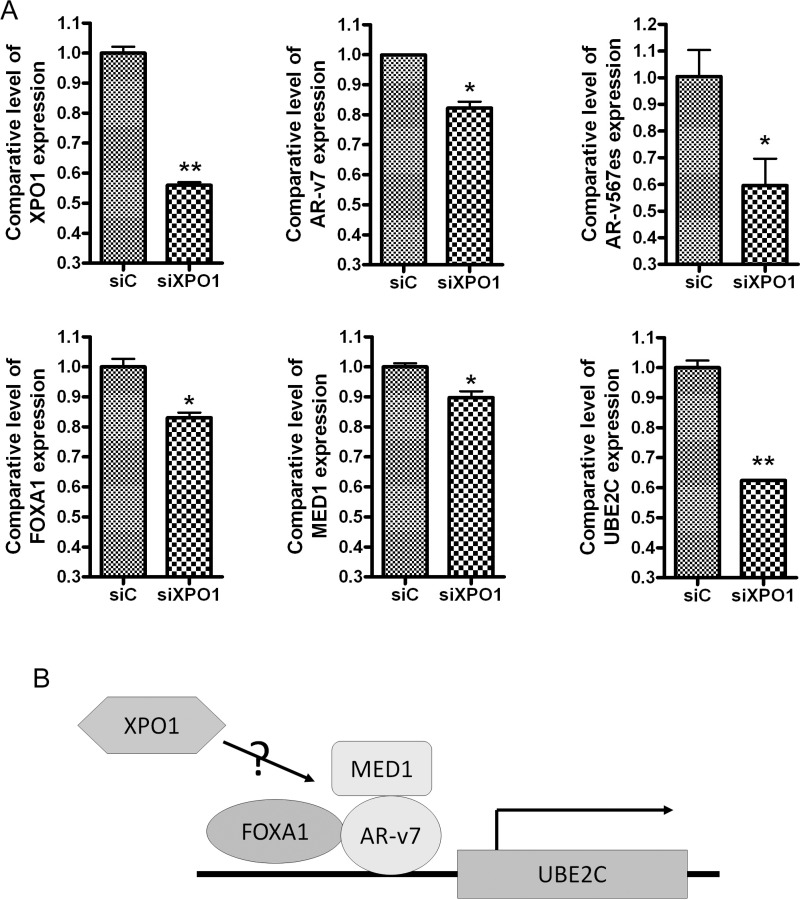
Silencing of XPO1 inhibits AR splice variants and their regulators (**A**) 22Rv1 cells were transfected with XPO1 siRNA. The expressions of XPO1, AR-v7, ARv567es, FOXA1, MED1 and UBE2C mRNA were tested by using real-time RT-qPCR. (**B**) The diagram showing possible regulatory mechanism underlying XPO1 regulated AR splice variant signaling.

Based on our observed connection between XPO1 and AR splice variants, we investigated whether XPO1 could regulate the expression of AR splice variants and their regulators. We transfected XPO1 siRNA into 22Rv1 PCa cells. We found that silencing XPO1 down-regulated the expression of AR splice variants (AR-v7 and ARv567es) and their regulators including FOXA1, MED1 and UBE2C (Figure 2A). FOXA1 and MED1 are co-regulators of AR splice variants [21, 22] and UBE2C is an AR splice variant target gene (Figure 2B) [23].

### SINE significantly inhibits XPO1, AR and AR splice variants

**Figure 3:**
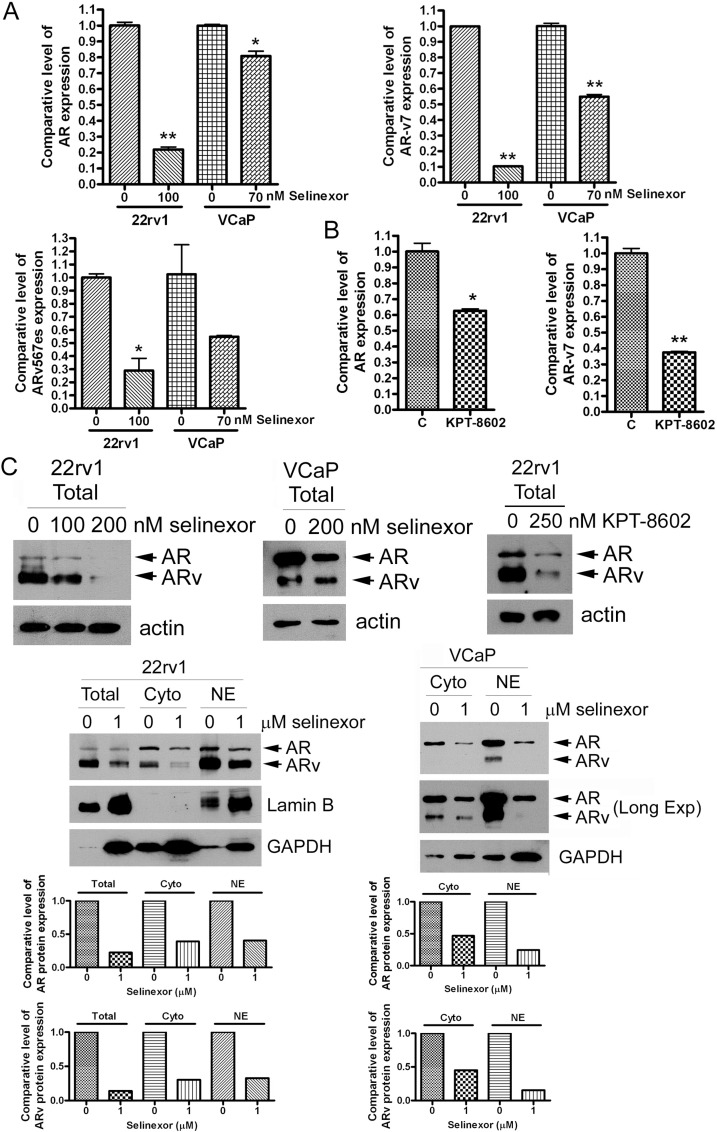
SINE significantly inhibits AR and AR splice variants 22Rv1 and VCaP cells were treated with 70–100 nM selinexor (**A**) or 200 nM KPT-8602 for 48 hours (**B**). The expressions of AR, AR-v7 and ARv567es mRNA were accessed by real-time RT-qPCR (^*^p < 0.05; ^**^p < 0.01). (**C**) 22Rv1 and VCaP cells were treated with 100–200 nM selinexor or 250 nM KPT-8602 for 72 hours and total proteins were extracted from these cells. 22Rv1 and VCaP cells were also treated with 1 μM selinexor for 24 hours and cytoplasmic and nuclear proteins were separately extracted from these cells. The expression levels of AR and ARv proteins were measured by using Western Blot analysis. The signal was quantified by using AlphaEaseFC and the expression level of AR and ARv was calculated and normalized by Lamin B for nuclear protein and GAPDH for total and cytoplasmic proteins.

Because silencing XPO1 using siRNA inhibited the expression of AR splice variants and their regulators, we tested the effects of SINE on expression of ARv and regulators. We treated 22Rv1 and VCaP cells with 70–100 nM selinexor or 200 nM KPT-8602 for 48 hours and measured the mRNA expression levels of AR, AR-v7 and ARv567es before and after SINE treatment. We found that SINE significantly inhibited the mRNA expression of AR, AR-v7 and ARv567es (Figure 3A and 3B). To confirm if the protein levels of AR and ARv were also decreased after the downregulation of AR and ARv mRNAs by SINE, we conducted Western Blot analysis. Western blot analysis also showed that SINE significantly decreased protein levels of AR and ARv in 22Rv1 and VCaP cells after SINE treatment for 72 hours (Figure 3C). Since the transcriptional effects of AR and AR splice variants occur in nucleus, we isolated and separated cytosol and nuclear proteins, and tested the expression levels of AR and ARv before and after SINE treatment. We found that SINE downregulated the levels of AR and ARv in both cytoplasmic and nuclear compartments (Figure 3C).

To confirm that the down-regulation of AR and ARv is the direct effect of SINE rather than the effects of cell growth inhibition, we treated 22Rv1 and VCaP cells with 250 nM selinexor for a short time (17 hours) when we did not observe cell growth inhibition. We found that selinexor treatment at short time also significantly inhibited the expression of AR and ARv at mRNA and protein levels (Supplementary Figure 2A and 2B). Moreover, selinexor significantly decreased the levels of AR and ARv in both cytoplasmic and nuclear compartments (Supplementary Figure 2B). These results suggest the direct inhibitory effects of selinexor on AR and AR splice variants.

### SINE inhibits the molecules which regulate ARv and its targets

**Figure 4:**
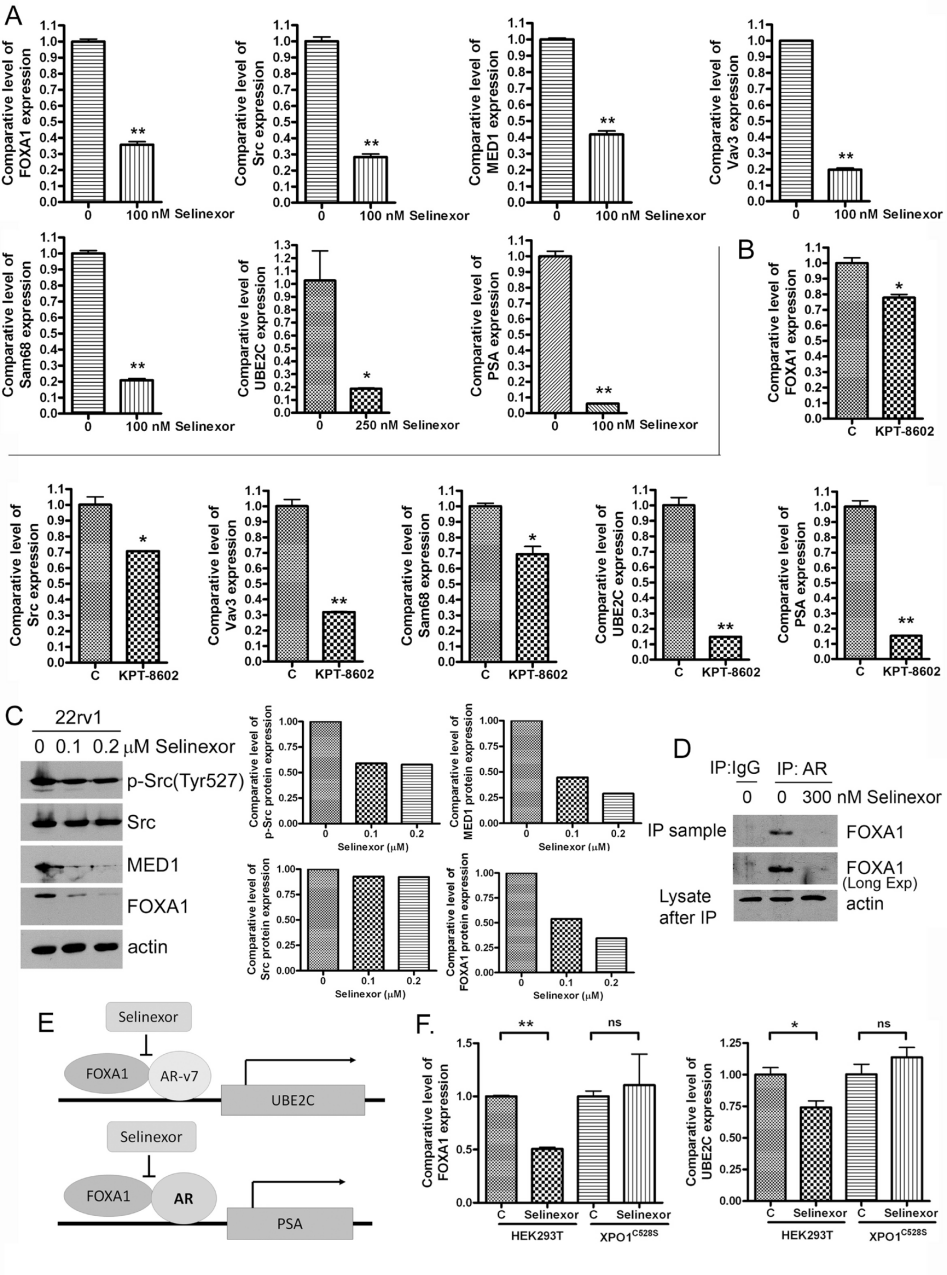
SINE inhibits regulators and targets of ARv and AR 22Rv1 cells were treated with 100 nM selinexor (**A**) or 200 nM KPT-8602 for 48 hours (**B**). The expressions of FOXA1, Src, MED1, Vav3, Sam68, UBE2C and PSA mRNAs were accessed by real-time RT-qPCR (^*^p < 0.05; ^**^p < 0.01). (**C**) 22Rv1 were treated with 100–200 nM selinexor for 72 hours and total proteins were extracted from these cells. The expression levels of p-Src, Src, MED1 and FOXA1 proteins were measured by using Western Blot analysis. The signal was quantified by using AlphaEaseFC and the expression level was calculated and normalized by actin. (**D**) The cell lysate from control and 300 nM selinexor treated 22Rv1 cells were immunoprecipitated with IgG or AR antibody which recognizes AR and ARv. Western Blot analysis was then conducted for testing FOXA1 binding to AR and ARv. (**E**) The diagram showing the regulatory mechanisms by which selinexor inhibits ARv, AR and their down-stream targets. (**F**) HEK293 XPO1 wild-type and mutant (C528S) cells were treated with 500 nM selinexor for 48 hours. The expressions of FOXA1 and UBE2C mRNAs were accessed by real-time RT-qPCR (^*^p < 0.05; ns: *p* > 0.05).

We conducted molecular experiments to further investigate the molecular mechanisms of SINE action on the regulation of AR and ARv. We found that in 22Rv1 PCa cells, SINE down-regulated mRNA and protein expression levels of FOXA1, Src, MED1, and Vav3 (Figure 4A–4C) which are known regulators of AR or ARv [21, 22, 24–26], suggesting that the inhibition of AR and ARv signaling by SINE could be mediated through these regulators. We also conducted immunoprecipitation assay to investigate the effects of selinexor on the interaction of FOXA1 and AR/ARv. We found that selinexor significantly inhibited AR/ARv binding to FOXA1 (Figure 4D), leading to the downregulation of transcription of UBE2C (Figure 4A and 4B), which is a downstream effector of AR splice variants (Figure 4E). Selinexor also down-regulated the expression of PSA (Figure 4A) which is a downstream target of AR (Figure 4E). These results suggest that the downregulation of ARv and AR signaling by SINE could be mediated through the inhibition of FOXA1/ARv or AR signal transduction.

To confirm the effect of SINE on FOXA1 and UBE2C is mediated through the regulation of XPO1, we treated HEK293 XPO1 wild-type and mutant (C528S) cells with SINE. XPO1 Cys528 is SINE binding site. The down-regulation of FOXA1 and UBE2C by SINE was observed in XPO1 wild-type cells and was not seen in XPO1 mutant (C528S) cells (Figure 4F), suggesting that the down-regulation of FOXA1 and UBE2C by SINE is mediated through XPO1 signaling.

### SINE regulates eukaryotic initiation factor 4E (eIF4E) to retain ARv RNA in nuclear compartment

**Figure 5:**
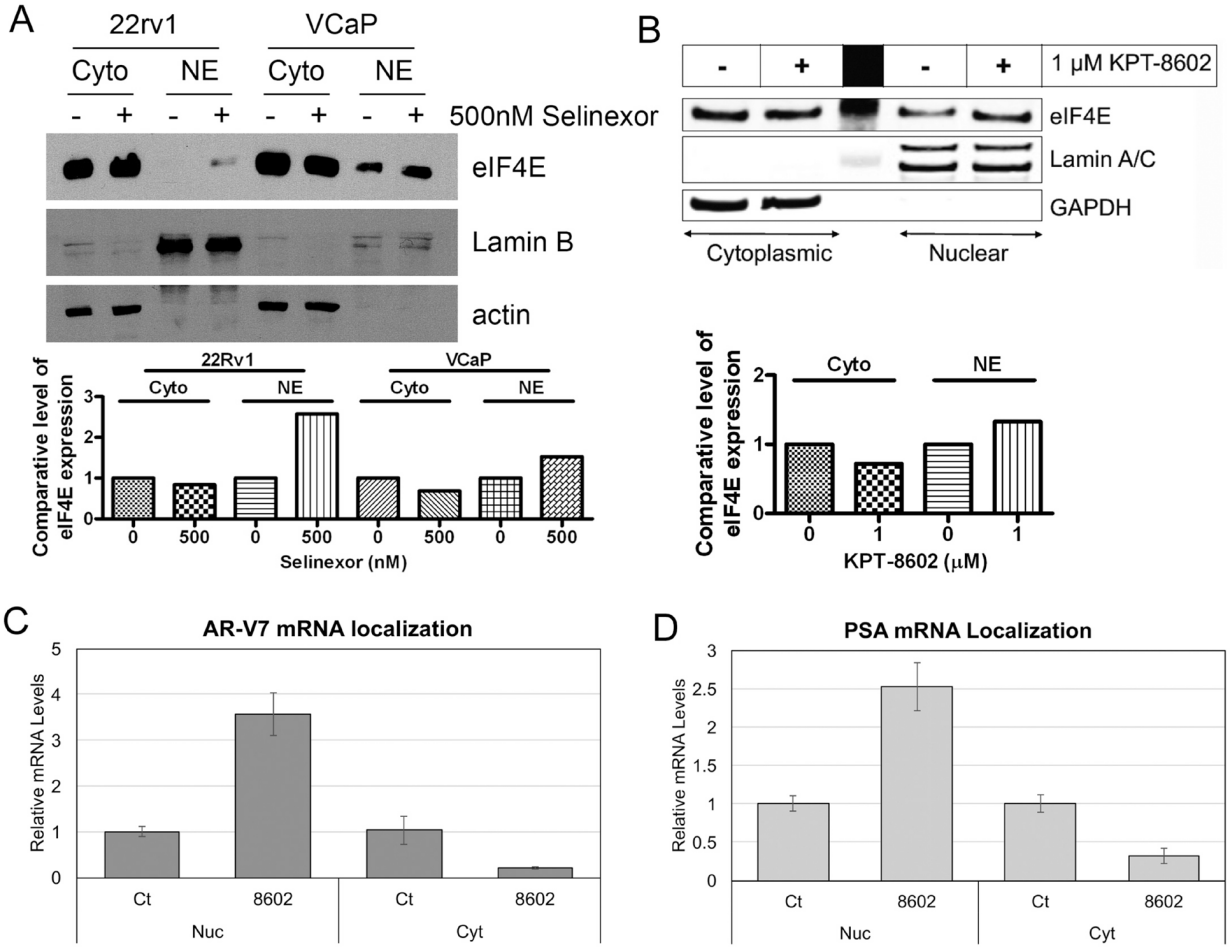
SINE regulates eIF4E to retain AR-v7 RNA in nuclear compartment 22Rv1 and VCaP cells were treated with 500 nM selinexor (**A**) or 1 μM KPT-8602 (**B**) for 24 hrs. Cytoplasmic and nuclear proteins were extracted and the expression of eIF4E was assessed by Western Blot analysis. The signal was quantified by using AlphaEaseFC and the expression level of eIF4E was calculated and normalized by Lamin for nuclear protein and actin or GAPDH for cytoplasmic protein. (**C** and **D**) RNA was isolated as nuclear (Nuc) and cytoplasmic (Cyt) fractions using the RNA Subcellular Isolation Kit. Real-time RT-qPCR was conducted for measurement of AR-v7 (C) and PSA (D) mRNAs.

To investigate the mechanism underlying the down-regulation of AR and AR-v7 by SINE, we measure the level of eIF4E in cytoplasmic and nuclear compartments before and after SINE treatment. eIF4E is responsible for the nuclear export and translation initiation of capped-dependent mRNAs [27, 28]. We found that SINE treatment retained eIF4E protein in nuclear compartment (Figure 5A and 5B), leading to nuclear retention of AR-v7 (Figure 5C) and PSA mRNA (Figure 5D). The nuclear retention of AR-v7 mRNA resulted in the reduction in the protein levels of ARv (Figure 3C). In addition, the deceased levels of AR and AR-v7 mRNA (Figure 3A and 3B) also contributed to the reduction of AR and ARv proteins observed in Figure 3C.

### SINE potentiates the anti-cancer activity of enzalutamide and abiraterone

**Figure 6:**
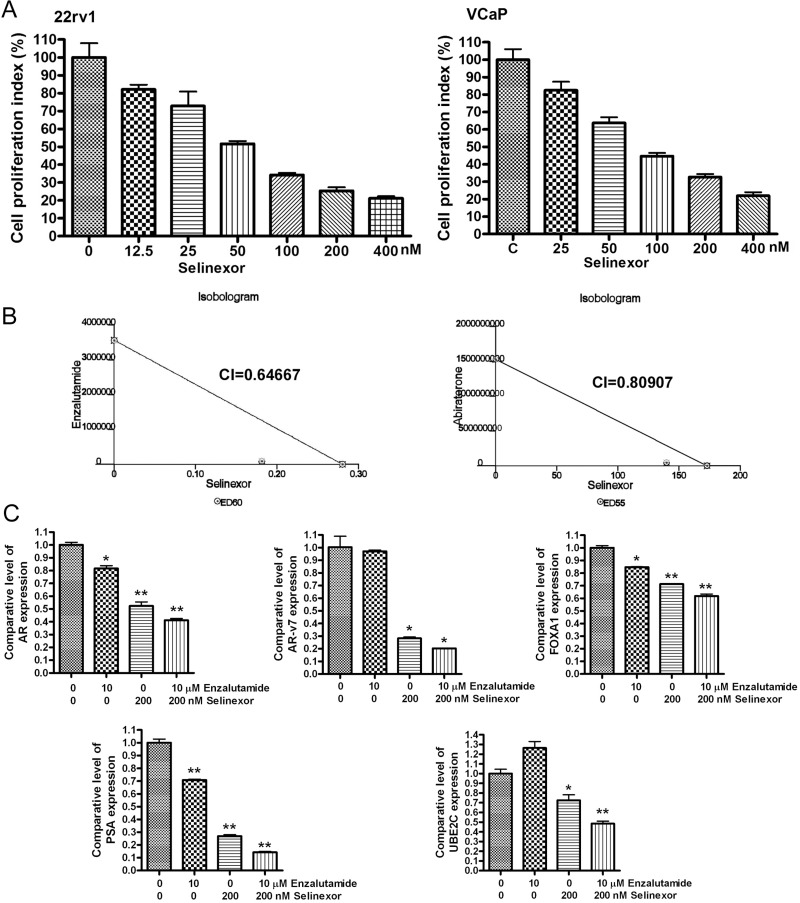
SINE potentiates the anti-cancer proliferation activity of enzalutamide and abiraterone (**A**) 22Rv1 and VCaP cells were treated with 12.5–400 nM selinexor for 72 hours. MTT assay was conducted to assess cell proliferation index. (**B**) 22Rv1 cells were treated with 50–200 nM selinexor, 5–20 μM enzalutamide, 5–20 μM abiraterone, or combination of selinexor with enzalutamide or abiraterone for 72 hours. MTT assay and isobologram analysis were conducted to assess the combination index (CI). (**C**) 22Rv1 cells were treated with 200 nM selinexor, 10 μM enzalutamide, or combination for 48 hours. The expression levels of AR, AR-v7, FOXA1, PSA and UBE2C mRNA were assessed by real-time RT-qPCR (^*^p < 0.05; ^**^p < 0.01).

Since we observed the inhibitory effect of SINE on AR/ARv signaling, we tested the anti-cancer activity of SINE. We treated 22Rv1 and VCaP PCa cells with different concentration of selinexor (from 12.5 nM to 400 nM). We found that selinexor significantly inhibited the proliferation of 22Rv1 and VCaP cells (Figure 6A), suggesting that the anti-cancer activity of SINE could be mediated through the down-regulation of AR/ARv signaling. Furthermore, we investigated whether selinexor could potentiate the anti-cancer activity of new anti-AR agents (enzalutamide and abiraterone) through the downregulation of AR and ARv. We treated 22Rv1 prostate cancer cells with selinexor (50–200 nM), enzalutamide (5–20 μM), abiraterone (5-20 μM), or combination of selinexor with enzalutamide or abiraterone. Isobologram analysis showed that selinexor combined with enzalutamide or abiraterone synergistically inhibited the cell proliferation (Figure 6B). Combination index (CI) at experimental conditions was less than 1 (Figure 6B), suggesting the synergistic effects of selinexor with enzalutamide or abiraterone on cell proliferation. Moreover, the combination treatment with selinexor and enzalutamide significantly inhibited the expression of AR, AR-v7, FOXA1, PSA and UBE2C (Figure 6C). These results demonstrate that SINE potentiates the anti-cancer proliferation activity of enzalutamide and abiraterone through the down-regulation of AR/ARv signaling.

### SINE inhibits tumor growth, potentiates anti-tumor activity of abiraterone and prolongs survival of a 22Rv1 xenograft

**Figure 7:**
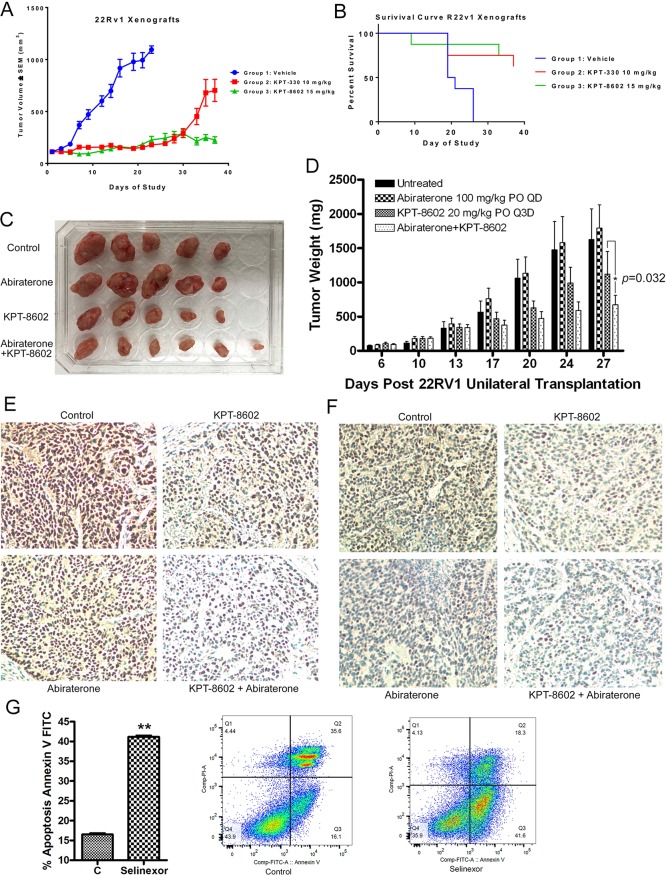
SINE inhibits tumor growth and prolongs survival of a 22Rv1 xenograft through retention of TSPs (**A**) %TGI on Day 16 was 84% and 87% by selinexor and KPT-8602, respectively, when compared to the vehicle. (**B**) Kaplan–Meier plot shows that vehicle treated mice have a median OS of 20 days while both SINE treatment groups have an undefined median OS at end of the study (Day 37). (**C** and **D**) KPT-8602 and abiraterone combination treatment significantly inhibited the growth of tumors in mice, showing decreased tumor size (C) and weight (D). The mouse tumor tissues with KPT-8602 and/or abiraterone were immunochemistry stained with anti-AR (**E**) or anti-ARv (**F**) antibodies. (**G**) 22Rv1 cells were treated with 250 nM selinexor for 48 hour. The apoptotic cell death was tested by Annexin V FITC assay.

To investigate the effects of SINE on tumor growth *in vivo*, we conducted animal experiments. We evaluated the efficacy of selinexor (10 mg/kg, QoDx3/week) and KPT-8602 (15 mg/kg, QDx5/week) in a 22Rv1 xenograft model in male CB.17 SCID mice. We found that %TGI on Day 16 was 84% and 87% by selinexor and KPT-8602, respectively, when compared to the vehicle (Figure 7A). Moreover, Kaplan–Meier plot analysis showed that vehicle treated mice had a median OS of 20 days while both SINE treatment groups had an undefined median OS at end of the study (Day 37) (Figure 7B). These results suggest that SINE significantly inhibits tumor growth and prologs survival of 22Rv1 xenograft.

Moreover, we evaluated the efficacy of combination treatment with KPT-8602 and abiraterone in the 22Rv1 xenograft model. We found that the combination treatment with KPT-8602 and abiraterone significantly inhibited the growth of 22Rv1 tumors in mice (Figure 7C and 7D) with significant suppression of AR (Figure 7E) and ARv (Figure 7F). These *in vivo* results are consistent with the *in vitro* data (Figure 6B) showing that SINE and abiraterone synergistically inhibited prostate cancer growth.

### SINE retains TSP in nucleus to inhibit cell proliferation and induce apoptosis

We further conducted immunohistochemistry analysis to demonstrate the retention of TSPs in nucleus of cancer cells *in vivo* after SINE treatment. The immunohistochemistry analysis of xenograft samples derived from 22Rv1 cells treated with vehicle control, selinexor or KPT-8602 showed decreased cell proliferation marker (Ki67) and increased apoptotic molecule (Cleaved Caspase 3) in samples treated with SINE compounds (Supplementary Figure 3). The increased cleaved Caspase 3 was consistent with increased apoptosis observed by Annexin V FITC assay (Figure 7G). Increased nuclear staining of TSPs Rb, p21, p53, APC and SMAD4 were also observed in samples treated with SINE compounds (Supplementary Figure 3).

## DISCUSSION

High expression of XPO1 has been found in many types of solid and hematopoietic malignant tumors including cervical cancer, prostate cancer, ovarian cancer, pancreatic cancer, gastric cancer, osteosarcoma, glioma, multiple myeloma, lymphoma and leukemia [14, 29–37]. The increased expression of XPO1 positively correlates with disease progression and reduced survival in these malignances. Although only one study has reported increased expression of XPO1 in prostate cancer tissue [14], the data from Oncomine database shows that more than 8 sets of mRNA expression profiles including XPO1 from PCa tissues (total 342 cases) and normal prostate gland tissues (total 170 cases) were tested by miRNA microarray. The Oncomine analysis tool reveals a higher expression of XPO1 in PCa tissues when compared to normal prostate gland tissues. The high expression of XPO1 in PCa tissues has been reported to be associated with a high Gleason score and bone metastasis [14]. These findings suggest the important role of XPO1 in the development and progression of PCa. More importantly, we found that the expression of XPO1 is correlated with high expression of AR-v7 which has been known as an important marker for disease progression and drug resistance in CRPC and PCa bone metastasis [6, 7, 9, 11]. We also found that silencing XPO1 down-regulates the expression of AR splice variants and their regulators. These results suggest that there is a relationship between XPO1 and AR splice variants. High expression of both XPO1 and AR-v7 could lead to constitutively activated AR signaling, CRPC development and progression, and anti-AR drug resistance.

Currently, the mechanisms underlying the XPO1 mediated regulation of AR and AR splice variants remains unknown. It has been reported that compounds targeting Src family kinases down-regulated expression and nuclear translocation of AR-v7 and inhibited ligand-independent transcription of AR target genes, suggesting that Src is an important upstream regulator of AR-v7 [26]. The RNA-binding protein Sam68 is another molecule which regulates the expression and activity of AR splice variants. Sam68 controls transcription of AR exon 3b, causing up-regulation of endogenous AR-v7 mRNA and AR-v7 protein [25]. Moreover, Sam68 activates ligand-independent transcriptional activity AR-v7 and induces the expression of AR-v7 target gene UBE2C [25], suggesting that Sam68 is an AR-v7 regulator. Vav3 is an AR coactivator; however, it also activates AR splice variants. It has been reported that Vav3 significantly promoted the transcriptional activity of AR splice variants including AR-v7 and ARv567es [24]. Knockdown of Vav3 or AR-v7 significantly inhibits ligand-independent AR activity, leading to suppression of PCa cell proliferation [24]. These findings demonstrate the regulatory effects of Vav3 on AR splice variants. Moreover, a recent study also demonstrates that FOXA1 regulates activity of AR splice variants in models of CRPC. It has been found that reduction of FOXA1 in AR-v7 containing 22Rv1 cells abrogated the oncogenic potential of AR splice variants [22]. Gene expression profiling showed that approximately 41% of AR splice variant transcriptome requires FOXA1, suggesting the regulatory effect of FOXA1 on AR splice variants [22]. Similar to FOXA1, MED1 also mediates ARv induced gene expression in the absence of ligand [21]. MED1 binds to ARv567es and promotes ARv567es induced UBE2C expression [21]. These results indicate that MED1 is a key regulator of ARv in the development and progression of CRPC. UBE2C is an important cell cycle gene enriched in CRPC. A study found that suppression of ligand-mediated AR signaling by enzalutamide or abiraterone caused increased expression of AR-v7 and ARv567es which are constitutively activated, leading to the increased expression of UBE2C and drug resistance [23]. In present study, we found that SINE down-regulated the above mentioned ARv regulations including Src, Sam68, Vav3, FOXA1 and MED1, leading to the decreased expression of ARv target gene UBE2C (Figure 4E). SINE also inhibited the expression of AR, resulting in the down-regulation of AR target genes such as PSA. The down-regulation of ARv target gene UBE2C and AR target genes by SINE suppresses PCa cell growth, which could be one of the molecular mechanisms by which SINE inhibits PCa growth and viability.

XPO1 is responsible for the exporting many TSPs from the nucleus to the cytoplasm, leading to a dysregulation of TSP localization and activity [38, 39]. In this study, we found that XPO1 inhibition by SINE compounds promotes the retention of TSPs including Rb, p21, p53, APC and SMAD4 in nucleus of PCa cells,. Interestingly, we also found that SINE retained eIF4E protein in nucleus. eIF4E plays a critical role in mRNA translation by binding the 5′-cap structure of the mRNA [40]. It controls the nuclear export and translation initiation of capped-dependent mRNAs [27, 28, 41]. Retention of eIF4E protein in nucleus by SINE results in the retention of AR-v7 and PSA mRNA in nucleus, leading to the reduced levels of AR-v7 and PSA protein, which could be one of the mechanisms underlying the down-regulation of AR, ARv and PSA by SINE. However, more mechanistic studies are needed to full understand how SINE regulates ARv mRNA.

In recent years, enzalutamide and abiraterone have been used as anti-AR drugs in AR-targeted therapies. Abiraterone is an inhibitor of CYP17 which is critical for androgen synthesis. By targeting CYP17, abiraterone decreases the level of circulating and intratumoral androgens, leading to the down-regulation of AR signaling [42]. Due to its steroidal structure, abiraterone also inhibits other molecules in AR pathway [42]. Enzalutamide is an inhibitor of AR signaling. By binding to the ligand-binding domain of AR, enzalutamide suppresses AR nuclear translocation and binding to DNA, leading to down-regulation of AR target genes [43, 44]. Treating PCa patients with abiraterone and enzalutamide improves response rates and overall survival. However, patients using abiraterone and enzalutamide commonly develop resistance to these agents [45, 46]. One of the key mechanisms involved in resistance to abiraterone and enzalutamide is the expression of constitutively activated AR splice variants that are refractory to anti-AR therapies [23, 45]. The anti-AR treatment-induced AR splice variants activate cell cycle genes such as UBE2C without requiring the presence of full length AR, leading to PCa survival and progression in castrate conditions [23]. In this study, we found that SINE significantly inhibited the expressions of XPO1, AR and AR splice variants, which make SINE a promising therapeutic for the treatment of PCa.

Because SINE inhibits ARv, SINE could sensitize PCa cells to anti-AR therapy. Indeed, we found that SINE *in vitro* and *in vivo* potentiated the anti-cancer activity of anti-AR agents, enzalutamide and abiraterone, by inhibition of XPO1 and AR splice variants. Our *in vitro* and *in vivo* findings are supported by clinical observations as well [20]. In this Phase II study (NCT02215161), fourteen patients were 60 mg flat dose twice a week (days 1 and 3), 3 weeks on, 1 week off with a median treatment duration of 13 weeks. At a median follow-up of 4 months, two patients (14%) had ≥50% prostate-specific antigen (PSA) decline, and seven patients (50%) had any PSA decline. Of eight patients with measurable disease at baseline, two (25%) had a partial response and four (50%) had stable disease as their best radiographic response. Despite these positive results, several patients experienced serious adverse events (SAEs). Nevertheless, these SAEs were unrelated to selinexor), and five patients (36%) experienced treatment-related grade3–4 AEs. The most common drug-related adverse events (AEs) of any severity were anorexia, nausea, weight loss, fatigue, and thrombocytopenia. The new generation SINE KPT-8602 has better tolerability. Therefore, we anticipate the outcome of our studies would lead to the introduction of KPT8602 in combination with conventional chemotherapeutics and AR-targeted therapy for the better treatment of PCa, especially CRPC.

## MATERIALS AND METHODS

### Cell lines, reagents and antibodies

22Rv1, VCaP, and LNCaP cells were purchased from American Type Culture Collection (ATCC, Manassas, VA) and maintained in RPMI1640 (Invitrogen, Carlsbad, CA) supplemented with 10% fetal bovine serum (FBS), 100 U/mL penicillin and 100 μg/mL streptomycin in a 5% CO_2_ atmosphere at 37°C. HEK293 XPO1 wild-type and mutant (C528S) were developed as described previously [47]. The cell lines have been tested and authenticated in a core facility of the Applied Genomics Technology Center at Wayne State University. The method used for testing was short tandem repeat (STR) profiling using the PowerPlex^®^ 16 System from Promega (Madison, WI). SINE including selinexor and KPT-8602 (Karyopharm Therapeutics, Newton, MA) were dissolved in DMSO to make a 1 mM stock solution. Anti-AR (N20) which recognizes both AR full length and AR splice variants (Santa Cruz, Santa Cruz, CA), anti-XPO1 (Santa Cruz), anti-FOXA1 (Novus Biologicals, Littleton, CO), anti-MED1 (Santa Cruz), anti-eIF4E (Cell Signaling, Danvers, MA), anti-Src (Cell Signaling), anti-p-Src(Tyr527) (Cell Signaling), anti-Lamin B (Invitrogen), and anti-β-actin (Sigma, St. Louis, MO) primary antibodies were used for Western Blot analysis.

### RNA isolation and mRNA real-time RT-qPCR

Total RNAs from PCa cell lines treated with SINE were extracted and purified by using the RNeasy Mini Kit and RNase-free DNase Set (QIAGEN, Valencia, CA) following the protocol provided by the manufacturer. Total RNAs from PCa tissues were isolated from formalin-fixed, paraffin-embedded tissue sections by using miRNeasy FFPE Kit (QIAGEN) following the protocol provided by the company. Retrospective archival PCa tissues were collected from patients who underwent routine radical prostatectomy at Karmanos Cancer Institute and obtained from Biospecimen Core of Karmanos Cancer Institute after obtaining institutional review board approval. The expression levels of AR, AR-v7, ARv567es, PSA, XPO1, FOXA1, MED1, Src, Vav3, Sam68, or UBE2C in selinexor or KPT-8602 treated or un-treated PCa cells and PCa tissues were analyzed by real-time RT-qPCR using High Capacity cDNA Reverse Transcription Kit and SYBR Green Master Mixture from Applied Biosystems (Waltham, MA). The sequences of primers used are listed in Table 1. The qPCR was initiated by 10 min at 95°C before 40 thermal cycles, each of 15 s at 95°C and 1 min at 60°C in a StepOnePlus real-time PCR system (Applied Biosystems). Data were analyzed according to the comparative Ct method and were normalized by actin and/or 18S rRNA expression in each sample.

**Table 1:** Sequences of primers used

Primers	Sequences	
AR	Forward	GACTTCACCGCACCTGATG
	Reverse	AATGGGCAAAACATGGTCCC
AR-v7	Forward	TGTCCATCTTGTCGTCTTCGG
	Reverse	TGCAATTGCCAACCCGGAAT
ARv567es	Forward	TTGTACACGTGGTCAAGTGGG
	Reverse	TGAACTGATGCAGCTCTCTCG
PSA	Forward	GTCCCGGTTGTCTTCCTCAC
	Reverse	CTCCCACAATCCGAGACAGG
XPO1	Forward	ACGAGGAAGGAAGGAGCAGT
	Reverse	CGAGCTGCATGGTCTGCTAA
FOXA1	Forward	ACCAGCGACTGGAACAGCTA
	Reverse	GTCATGTTGCCGCTCGTAGT
MED1	Forward	GCTTGTGCGTCAAGTCATGG
	Reverse	TGAGATGAGAGCCCAGTCCA
Src	Forward	TGTTCGGAGGCTTCAACTCC
	Reverse	TGTGTTGTTGACAATCTGGAGC
Vav3	Forward	GGACTCGGCTCAGGTGTTCG
	Reverse	GCCCGGAGGTTGTTAAGCAG
Sam68	Forward	TGAGAGACAAAGCCAAGGAGG
	Reverse	CTCACATGGGGGTCCAAAGA
UBE2C	Forward	TCCTGTCTCTCTGCCAACGC
	Reverse	TTGTCTGATTCAGGGAAGGCA
actin	Forward	GCACAGAGCCTCGCCTT
	Reverse	TCATCATCCATGGTGAGCTG
18 S	Forward	GCAATTATTCCCCATGAACG
	Reverse	GGCCTCACTAAACCATCCAA

### Separation of cytoplasmic and nuclear RNA

RNA from selinexor or KPT-8602 treated 22Rv1 cells was isolated as cytoplasmic and nuclear fractions using the RNA Subcellular Isolation Kit. The AR-v7 and PSA mRNA levels in cytoplasmic and nuclear compartments were measured by real-time RT-qPCR using the following taqman assays (Hs00171172_m1 AR, Hs00907242_m1 AR, and Hs02576345_m1 KLK3).

### Preparation of total, cytoplasmic and nuclear protein lysates

For total protein extraction, PCa cells treated or untreated with SINE were lysed in RIPA buffer and protein concentration was measured using BCA protein assay (PIERCE, Rockford, IL). For cytoplasmic and nuclear protein extraction, PCa cells with and without SINE treatment were harvested and incubated in ice-cold cell lysis buffer (10 mM HEPES pH7.9, 10 mM KCl, 0.1 mM EDTA, 0.1 mM EGTA, 1 mM DTT, 0.5 mM PMSF, 1X protease inhibitor cocktail) on ice. After 15 minutes, NP-40 was added to the cell suspension at a final concentration of 0.3% and the samples were vortexed vigorously for 20 seconds. After centrifugation, the supernatant was saved as cytoplasmic protein and the nuclear pellet was incubated in ice-cold nuclear extraction buffer (20 mM HEPES pH7.9, 0.4 M NaCl, 1 mM EDTA, 1 mM EGTA, 1 mM DTT, 0.5 mM PMSF, 1X protease inhibitor cocktail) on ice for 30 minutes. After centrifugation, the supernatant was saved as nuclear protein and protein concentration was measured using BCA Protein Assay (PIERCE).

### Immunoprecipitation

Total lysate (300 μg) from each sample were diluted and subjected to immunoprecipitation using 5 μg of anti-AR (N20) antibody or normal rabbit IgG (Cell Signaling). The lysate and antibody mixtures were incubated overnight at 4°C with rotation. After adding 30 μl of Protein G Agarose (Santa Cruz) and incubation for 2 hour, the samples were centrifuged. The agarose pellet was then washed three times, resuspended in 50 μl of Laemmli buffer with 2-mercaptoethanol, and boiled for 5 minutes. Boiled samples were centrifuged and supernatant was used for Western Blot analysis.

### Western blot analysis

Western Blot analysis was conducted to measure the alterations in the protein expression of genes. Briefly, the total, cytoplasmic, and nuclear proteins were subjected to 10 or 14% SDS-PAGE, and electrophoretically transferred to nitrocellulose membrane. The membranes were incubated with specific primary antibodies, and subsequently incubated with secondary antibody conjugated with peroxidase (Bio-rad, Hercules, CA). The signal was detected using the chemiluminescent detection system (PIERCE) and quantified by using AlphaEaseFC (Alpha Innotech, FL).

### Inhibition of XPO1 expression by siRNA in PCa cells

22Rv1 PCa cells were seeded in a 6 well plate (3 × 10^5^ cells per well) and incubated at 37°C for 24 hours. The cells were then transfected with XPO1 siRNA (Santa Cruz) or control siRNA by DharmaFact Transfection Reagent (Dharmacon) for 72 hours. Then, the total RNA was extracted and subjected to mRNA RT-qPCR for testing AR-v7, ARv567es, FOXA1, MED1, UBE2C and XPO1 expression.

### Growth inhibition and isobologram assay

22Rv1 and VCaP cells were treated with 50–200 nM selinexor, 5–20 μM enzalutamide, 5–20 μM abiraterone, or combination of selinexor with enzalutamide or abiraterone for 72 hours. Then, the cells were subjected to cell proliferation assay using MTT [3-(4,5-dimethylthiazol-2-yl)-2,5-diphenyltetrazolium bromide]. The spectrophotometric absorbance of the samples was determined by using a plate reader SynergyHT (BioTek, Winooski, WI) at 570 nm. The combination index (CI) value and isobologram were calculated and created by using CalcuSyn software (Biosoft, Cambridge, UK).

### Quantification of apoptosis by annexin V FITC assay

Cell apoptosis was detected using Annexin V FITC (Biovision Danvers MA) according to the manufacturer's protocol as we reported previously [48]. PCa cells were treated with 300 nM selinexor for 72 hrs. At the end of treatment, cells were trypsinized and equal numbers were stained with Annexin V and Propidum Iodide. The stained cells were analyzed using a Becton Dickinson flow cytometer at the Karmanos Cancer Institute Flow Cytometry Core.

### Animal studies

The study was designed to evaluate the efficacy of selinexor (10 mg/kg, QoDx3/week) and KPT-8602 (15 mg/kg, QDx5/week) in a 22Rv1 prostate cancer xenograft model in CB.17 SCID mice. Mouse weights and tumor size were monitored three times a week. Each mouse was euthanized at the end of study (Day 35) or when tumor volume reached 1000 mm^3^, whichever comes first. Tumor were collected and fixed in 10% formalin for histopathology analysis.

The effects of combination treatment with KPT-8602 and abiraterone was also evaluated in the 22Rv1 prostate cancer xenograft model in ICR-SCID mice. Six days after 22Rv1 transplantation, mice were randomized into 4 groups: Untreated (*n* = 5), abiraterone acetate treated (*n* = 5), KPT-8602 treated (*n* = 5) and combination treatment (*n* = 6). Abiraterone (Selleckchem) was administered orally each day at the dose of 100 mg/kg for 3 weeks. KPT-8602 was administered orally at 20 mg/kg twice a week for a total of 7 doses. All mice were followed for measurement of S.C. tumors and observed for changes in body weight and any side effects. All tumors were collected at 24 hours after last dose of combination treatment and tumor picture was taken.

### Immunostaining

A tissue microarray (TMA) was constructed with tumor samples collected from 22Rv1 mouse prostate cancer model. Paraffin sections of the TMA were processed and stained with antibodies using a Biogenex I6000 automated stainer. Digital images of the slides were obtained through an Aperio AT Turbo scanner at 20×. The following antibodies were used for immunohistochemistry staining: Cleaved Caspase 3 (Cell Signaling Technology, 9661), p21 (Cell Signaling Technology, 2947), APC (Abcam, ab15270), Ki67 (Cell Marque, 275R-18), Rb (Abcam, ab181616), p53 (Santa Cruz, sc-126), SMAD4 (Santa Cruz, sc-7966), AR (Abcam, ab105225, and ARv (Abcam, ab198394).

### Statistics

Wherever appropriate, the data were subjected to a Student's *t*-test using GraphPad Prism software (La Jolla, CA). *p* < 0.05 was considered statistically significant.

## SUPPLEMENTARY MATERIALS FIGURES


