# Supplementary material for: Down-regulation of AR splice variants through XPO1 suppression contributes to the inhibition of prostate cancer progression
Source: Oncotarget. 2018 Oct 19;9(82):35327–42. doi: 10.18632/oncotarget.26239 (PMC6219671; doi:10.18632/oncotarget.26239)
Supplement: Supplementary file 1 [file oncotarget-09-35327-s001.pdf]

Down-regulation of AR splice variants through XPO1 suppression contributes to the inhibition of prostate cancer progression

SUPPLEMENTARY MATERIALS

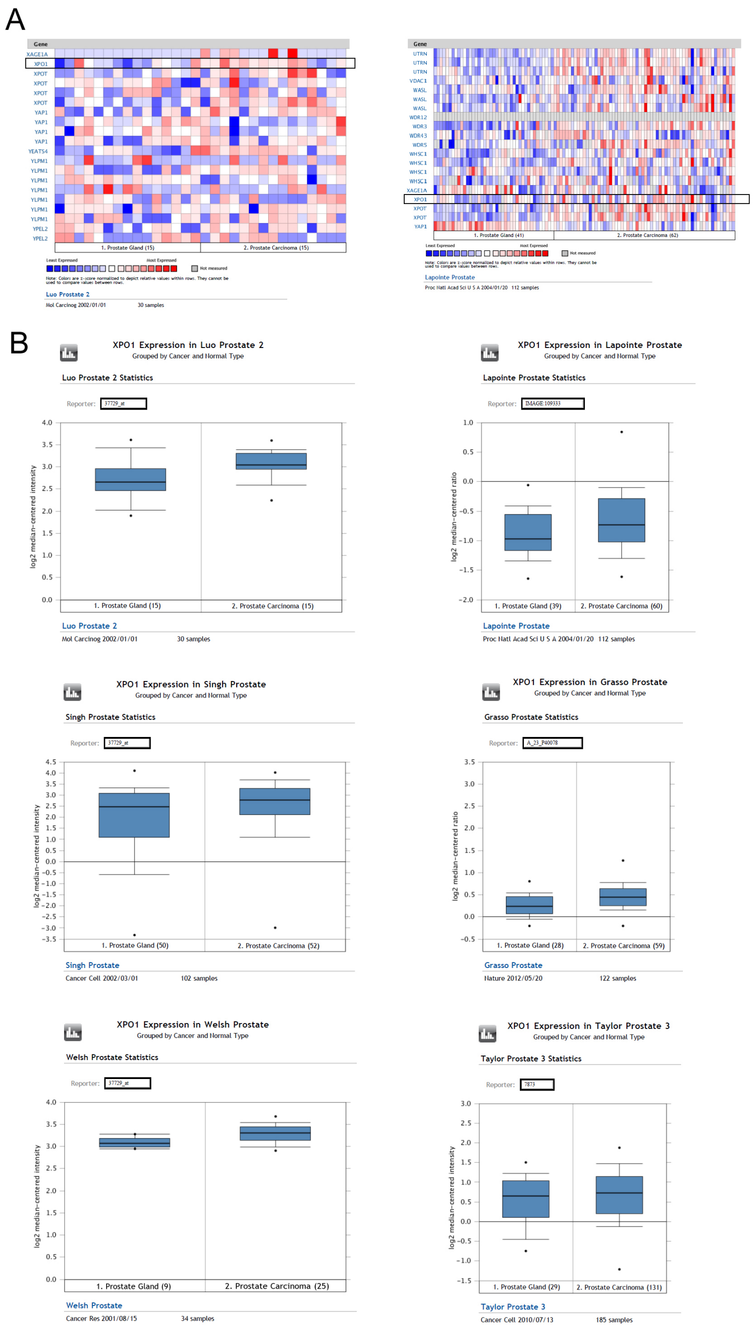

Supplementary Figure 1: High expression of XPO1 in PCa tissues compared to normal prostate gland. The mRNA microarray data sets from Oncomine database were analyzed for XPO1 mRNA expression in PCa tissues and normal prostate gland. Heatmaps (**A**) and box plots (**B**) show higher level of XPO1 expression in PCa tissues compared to normal prostate gland.

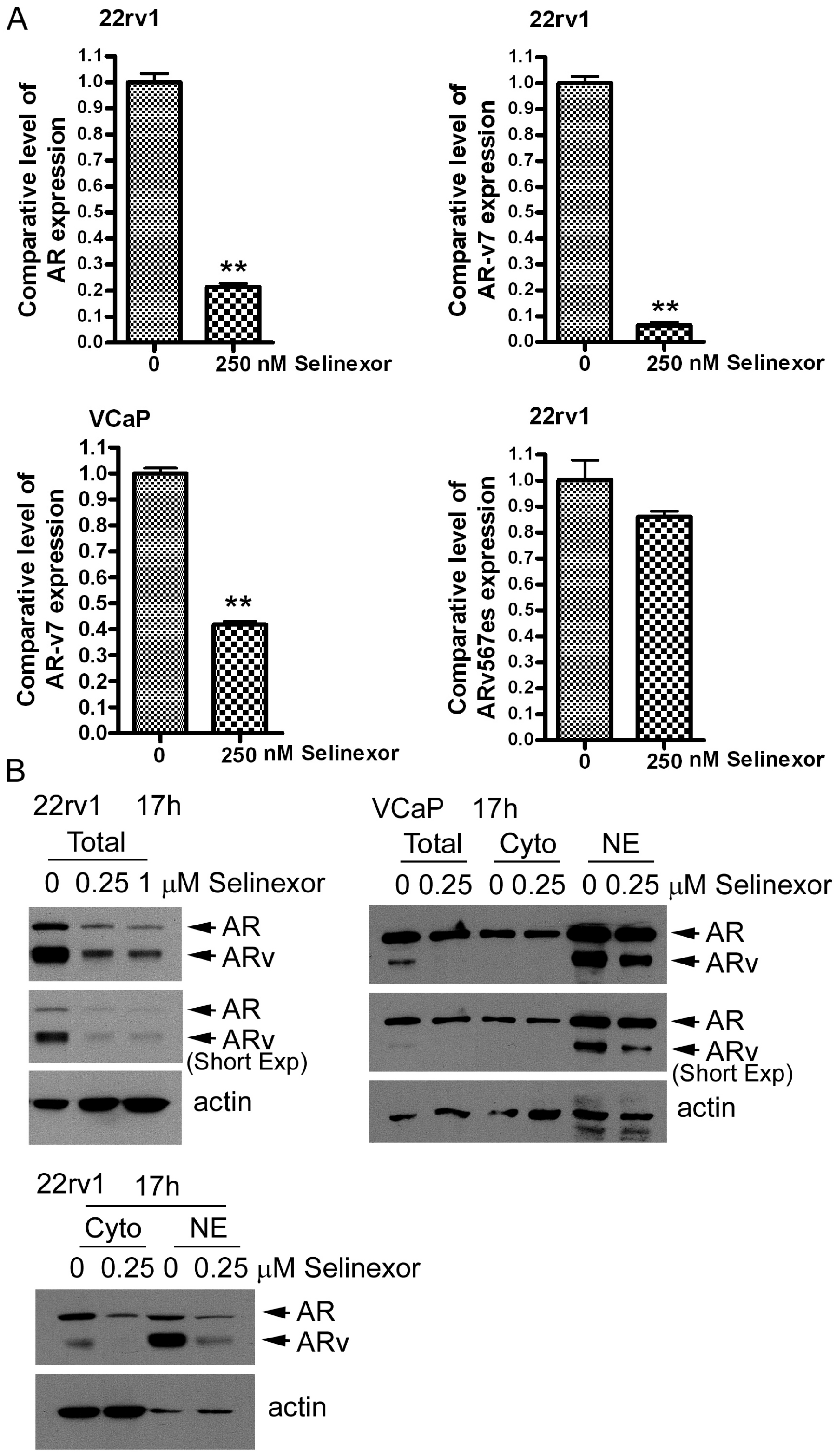

Supplementary Figure 2: SINE also significantly inhibits AR and ARv in short time treatment. 22Rv1 cells were treated with 250 nM selinexor for 17 hours. (**A**) The expression of AR, AR-v7, ARv567es and XPO1 mRNA were accessed by real-time RT-qPCR ($^{*}p < 0.05$; $^{**}p < 0.01$). (**B**) 22Rv1 cells were treated with 250 and 1000 nM selinexor for 17 hours. Total, cytoplasmic and nuclear proteins were separately extracted from these cells. The expression levels of AR and ARv proteins were measured by using Western Blot analysis.

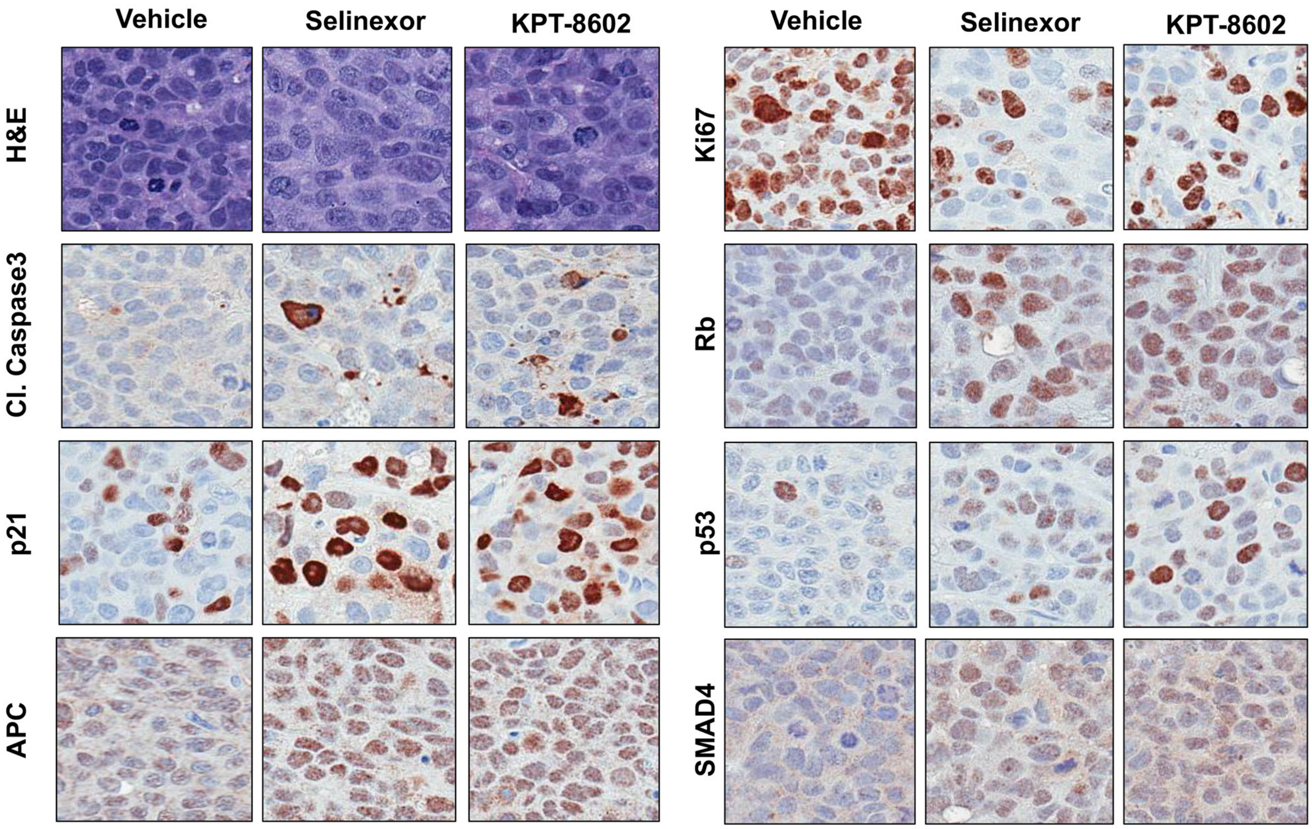

Supplementary Figure 3: SINE retains TSP in nucleus to inhibit cell proliferation and induce apoptosis. Immunohistochemistry analysis of xenograft samples derived from 22Rv1 cells treated with vehicle control, selinexor or KPT-8602. Decreased cell proliferation. (Ki67) and increased apoptosis (Cleaved Caspase 3) was observed in samples treated with SINE compounds. Increased nuclear staining of tumor suppressor proteins Rb, p21, p53, APC and SMAD4 were also observed in samples treated with SINE compounds.
